# Predicting Psychological Flourishing Among Psychologists: Integrating Self-Compassion, Traditional Regression, and Machine Learning Approaches

**DOI:** 10.3390/healthcare14182924

**Published:** 2026-09-09

**Authors:** Rania Maher Alhalawany, Rahaf Fahad AlNufaie, Yahya Mubark Khatatbeh

**Affiliations:** 1 Department of Health Sciences, College of Health and Rehabilitation Sciences, Princess Nourah bint Abdulrahman University, P.O. Box 84428, Riyadh 11671, Saudi Arabia; 2 Department of Psychology, College of Social Sciences, Imam Mohammad Ibn Saud Islamic University (IMSIU), P.O. Box 5701, Riyadh 11432, Saudi Arabia

**Keywords:** psychological flourishing, self-compassion, psychologists, mental health, machine learning, random forest, support vector regression, psychological well-being

## Abstract

**Background**: Psychological flourishing is a key indicator of optimal mental health and professional well-being, particularly among psychologists who are routinely exposed to emotionally demanding clinical environments. Although self-compassion has consistently been associated with positive psychological outcomes, few studies have integrated traditional statistical methods with machine learning approaches to predict psychological flourishing among psychologists. **Objective**: This study aimed to examine the relationship between self-compassion and psychological flourishing among psychologists in Saudi Arabia, identify the unique contribution of self-compassion dimensions, evaluate the predictive performance of supervised machine learning models, and compare their performance with traditional multiple linear regression. **Methods**: A cross-sectional correlational design was employed, involving 224 psychologists practicing in Saudi Arabia. Participants completed the Self-Compassion Scale and the Flourishing Scale. Descriptive statistics, Pearson’s correlation, and multiple linear regression analyses were performed using IBM SPSS Statistics version 29.0. In addition, Random Forest Regression and Support Vector Regression (SVR) models were implemented in Python using scikit-learn version 1.8.0 to predict psychological flourishing based on self-compassion dimensions together with demographic and professional characteristics. Model performance was evaluated using the coefficient of determination (R^2^), root mean square error (RMSE), and mean absolute error (MAE). **Results**: Overall, self-compassion was positively associated with psychological flourishing (r = 0.627, *p* < 0.001). Multiple linear regression showed that self-kindness had a significant positive independent association with psychological flourishing (β = 0.292, *p* = 0.001), whereas over-identification had a significant negative independent association (β = −0.206, *p* = 0.009). The regression model explained 40.5% of the variance in psychological flourishing (R^2^ = 0.405, *p* < 0.001). In the held-out test-set comparison using the same predictor set, predictive performance was similar across multiple linear regression (R^2^ = 0.273; RMSE = 3.583; MAE = 2.849), Random Forest Regression (R^2^ = 0.282; RMSE = 3.562; MAE = 2.771), and Support Vector Regression (R^2^ = 0.272; RMSE = 3.587; MAE = 2.768), with no substantial predictive advantage of the machine-learning models over the linear benchmark. **Conclusions**: Self-compassion, particularly self-kindness and over-identification, was significantly correlated with psychological flourishing among psychologists. Machine-learning models demonstrated predictive performance comparable to traditional regression, with no substantial predictive advantage over the linear benchmark, indicating that increased model complexity did not improve out-of-sample prediction in the present sample.

## 1. Introduction

Positive psychology has revolutionized modern psychology by shifting its focus from mental illness to optimal functioning, resilience, and well-being [1,2]. This perspective conceptualizes mental health not merely as the absence of psychopathology but also as the development of psychological protective resources that help people flourish in the emotional, psychological, and social domains [3,4]. Flourishing is one of the most comprehensive indicators of positive mental health, a state that has recently been advanced theoretically by synthesizing hedonic well-being (happiness and life satisfaction) with eudaimonic well-being (meaning, purpose, personal growth, competence, and positive interpersonal relationships) [5,6]. Flourishing has become much more important over the last decade, spreading from the field of psychology to public health, education, and occupational health. Flourishing is increasingly used as an important indicator of sustainable well-being and quality of life in these fields [7,8]. Flourishing has been conceptualized in recent reviews as a multidimensional construct assessed by multiple validated theoretical models. There is increasing agreement that mental health should be conceptualized as positive functioning rather than simply the absence of psychological distress [6,9]. This is particularly relevant in countries with comprehensive strategies for health and social transformation. Saudi Vision 2030 aims to improve the quality of life, psychological well-being, and community resilience and to develop mental health services through evidence-based initiatives [10].

Psychologists are among the most psychologically vulnerable professional groups, as their work involves constant contact with the emotional suffering of clients, traumatic experiences, and complicated interpersonal problems. Psychologists, compared to many other healthcare professionals, are expected to regulate their emotional reactions. At the same time, psychologists are expected to maintain high levels of empathy, emotional regulation, ethical decision-making, and therapeutic effectiveness. Such long-lasting emotional involvement makes them more vulnerable to occupational stress, emotional exhaustion, burnout, compassion fatigue, and secondary traumatic stress, which could negatively impact the clinician’s well-being and the quality of psychological care provided to clients [11,12,13]. Systematic evidence has recently indicated that psychologists often encounter moderate to high levels of occupational stress. Recent evidence further suggests that clinically meaningful stress symptoms are common among psychologists, underscoring the need for preventive strategies to support psychological resilience and sustainable professional functioning [14].

The consequences of prolonged occupational stress extend beyond individual mental health and influence professional performance, therapeutic relationships, and ethical practices. Burnout has been associated with poor concentration, emotional detachment, reduced empathy, an impaired therapeutic alliance, and an increased risk of clinical errors and professional attrition. Furthermore, compassion fatigue and secondary traumatic stress may hinder psychologists’ ability to adequately meet the emotional needs of their clients, which could compromise the quality of treatment and sustainability of their professional practice [11,12,15]. Recent reviews have identified self-care, self-compassion, work–life balance, and professional autonomy as potentially important protective factors against burnout and psychological distress among psychologists [16].

These findings emphasize the value of fostering positive psychological resources rather than focusing solely on risk reduction. Therefore, growing evidence supports a paradigm shift from treating occupational distress after its occurrence to identifying protective psychological mechanisms that enable psychologists to maintain well-being despite working in emotionally demanding environments. This perspective is fully aligned with positive psychology, which emphasizes the cultivation of personal strengths and adaptive psychological resources capable of enhancing resilience, professional satisfaction, and long-term flourishing. Research has identified the factors that promote flourishing among psychologists as a major research priority with implications for workforce sustainability, the quality of mental health services, and health policy [17,18].

Self-compassion has been identified as one of the most powerful protective resources in positive psychology and mental health research. Neff [19] originally conceived of self-compassion as a healthy and adaptive way of relating to oneself in the face of failure, suffering, and personal inadequacy, encouraging people to respond to distress with kindness, balanced awareness, and an understanding of the shared nature of human imperfection rather than self-criticism or avoidance [20,21]. The construct is composed of six interrelated dimensions (self-kindness, common humanity, mindfulness, self-judgment, isolation, and over-identification), which collectively represent the tendency of an individual to adaptively manage difficult emotional experiences. Although initially examined primarily within clinical psychology, self-compassion has increasingly become a central construct in occupational health, educational psychology, and positive psychology because of its consistent association with resilience, emotional regulation, and psychological well-being [22,23].

Growing empirical evidence indicates that self-compassion is associated with a broad range of positive psychological outcomes while simultaneously protecting against emotional distress and maladaptive coping. Recent systematic reviews and meta-analyses have consistently demonstrated that individuals with higher self-compassion report lower levels of depression, anxiety, stress, rumination, self-criticism, and experiential avoidance as well as higher levels of emotional well-being, resilience, optimism, and adaptive coping strategies. Moreover, a large systematic review combining evidence from 113 empirical studies concluded that the beneficial effects of self-compassion are mainly mediated by adaptive emotion regulation, less repetitive negative thinking, and mindfulness-based coping strategies [19,24].

Recent evidence also suggests that self-compassion plays a particularly important role among healthcare professionals and mental health practitioners who are regularly exposed to emotionally demanding clinical situations. Research indicates that compassion-based interventions have a positive effect on mental health, burnout, self-criticism, shame, and professional resilience, all of which can support long-term psychological functioning in helping professions. Thus, self-compassion has gained increasing recognition as a modifiable protective resource that can be further enhanced through evidence-based interventions and thus constitutes an important target for psychological prevention programs aimed at the promotion of flourishing and sustainable professional well-being [19,25,26,27].

There is increasing evidence that self-compassion is one of the most important resources for psychological flourishing. People who have more self-compassion also tend to report higher levels of life satisfaction, optimism, positive affect, resilience, meaning in life, and general psychological well-being and lower levels of depression, anxiety, stress, loneliness, and emotional exhaustion [6,20]. These findings support the view that self-compassion facilitates adaptive emotion regulation, reduces maladaptive self-evaluative processes, and enhances people’s ability to cope effectively with adversity, thus leading to flourishing. In addition, flourishing has increasingly been conceptualized as a function of multiple interacting psychological resources rather than as a single protective factor, with self-compassion being one of the most consistent predictors across different populations and cultural contexts.

Recent empirical studies have extended this evidence by demonstrating that self-compassion not only correlates positively with flourishing but also exerts indirect effects through several psychological mechanisms. For example, self-compassion leads to optimal psychological functioning through hope, psychological flexibility, mindfulness, emotional regulation, and resilience. A recent study of 842 university students found a significant positive association between self-compassion and psychological flourishing, with hope as a mediator and emotion regulation as a buffer. This highlights the complexity of the pathways linking self-compassion to positive mental health outcomes. Recent systematic reviews have also shown that increases in self-compassion are consistently linked with a wide range of improvements in psychological well-being in clinical, educational and community settings. These advances notwithstanding, the current literature has several limitations. First, most previous studies used traditional statistical methods, especially correlation and multiple linear regression, to analyze the relationship between self-compassion and flourishing. These methods are useful in identifying important relationships and predictors but provide limited information about how well the psychological variables predict when applied to new data. Second, few studies have specifically examined these relationships among practicing psychologists who experience unique psychological demands from their profession.

An important methodological consideration in machine-learning research is whether predictive performance can be generalized beyond the data used for model development. Internal validation provides an initial assessment of model performance, whereas evaluation using independent data offers stronger evidence regarding reproducibility and generalizability. This distinction is particularly important in health-related applications, where model performance may vary across populations, settings, and data distributions [28,29]. Sample size also plays a critical role in the stability of predictive models. Models developed from limited samples may be more vulnerable to overfitting and unstable performance estimates, particularly as model complexity increases. Recent methodological research therefore emphasizes considering sample size in relation to the number of predictors and model complexity, and interpreting predictive performance cautiously when the available development sample is limited [30,31].

Recent methodological guidance further emphasizes that predictive performance estimated within the development sample should be distinguished from model generalizability to independent data. Internal validation procedures, including train–test splitting and cross-validation, provide important evidence regarding predictive performance within the available sample, but external evaluation in independent samples is needed to establish whether model performance generalizes to new populations and settings. This distinction is particularly relevant when machine-learning models are developed using relatively limited samples, where model complexity may not necessarily translate into improved out-of-sample performance [32,33,34].

Thus, there is a need for research that not only explains but also predicts for professional populations whose psychological well-being carries direct implications for the quality of mental health services [35,36].

Moreover, prior studies have shown that self-compassion is highly correlated with psychological well-being [20,37]. However, most studies have used traditional statistical methods, especially correlation and multiple linear regression analyses, to assess these relationships. These methods are useful for variable selection and explaining variance but are limited in their ability to model complex non-linear relationships and to evaluate out-of-sample predictive performance. Random Forest Regression and Support Vector Regression (SVR) were selected because they provide complementary approaches to nonlinear prediction: Random Forest can capture complex interactions and nonlinear relationships through an ensemble of decision trees, whereas SVR can model nonlinear patterns in relatively small datasets through kernel-based learning while controlling model complexity. These methods have been shown to hold potential for psychological assessment, mental health prediction, and clinical decision support [38,39,40].

Recent applications of machine learning in healthcare further demonstrate the value of comparing multiple modelling approaches while emphasizing rigorous out-of-sample evaluation. Machine-learning methods have increasingly been applied to psychological and health-related outcomes, including depression and anxiety prediction, where model stability may be affected by inaccuracies inherent in self-reported data. More recent work has also applied regression-based machine-learning approaches to predict health-related quality of life, highlighting the importance of training–test separation, cross-validation, and comparison between linear and nonlinear models. Collectively, these developments support the use of machine learning as a complementary predictive framework while underscoring the need for cautious interpretation, particularly when models are developed from self-reported and cross-sectional data [38,41].

Despite this progress, little research has employed machine learning to predict psychological flourishing, particularly among practicing psychologists. To the best of our knowledge, relatively few studies have integrated traditional statistical and machine-learning approaches to examine psychological flourishing among psychologists. Addressing this gap may provide a more comprehensive understanding of psychological flourishing and offer preliminary insights that could inform future research on supportive and preventive mental health strategies.

This study investigated the association between self-compassion and psychological flourishing among psychologists in Saudi Arabia. There is increasing evidence of an association between self-compassion and positive psychological functioning. In addition, this study extends previous research by combining traditional statistical analysis and supervised machine learning techniques. The current study investigated the relationship between self-compassion and psychological flourishing, the unique contribution of each dimension of self-compassion, and the predictive performance of Random Forest Regression and Support Vector Regression (SVR) models compared to traditional multiple linear regression. This work aims to examine the potential application of machine learning in positive psychology by integrating descriptive, inferential, and predictive analytical approaches, while providing a cautious assessment of the extent to which data-driven methods may complement conventional statistical analyses of psychological well-being [38,39,40]. Accordingly, this study addressed the following research questions:

RQ1. Is there a significant relationship between self-compassion and psychological flourishing among psychologists?

RQ2. Which dimensions of self-compassion are independently associated with psychological flourishing among psychologists when examined using traditional multiple regression analysis?

RQ3. What is the predictive performance of machine-learning models in predicting psychological flourishing among psychologists using self-compassion dimensions and demographic/professional characteristics?

RQ4. How does the predictive performance of machine learning models compare with that of traditional multiple linear regression in predicting psychological flourishing among psychologists?

## 2. Materials and Methods

### 2.1. Study Design, Setting, and Participants

This cross-sectional correlational study examined the associations between self-compassion and psychological flourishing among practicing psychologists in Saudi Arabia. It also examined the independent associations of the self-compassion dimensions with psychological flourishing using traditional multiple regression analysis and evaluated their predictive contribution using supervised machine-learning approaches. The subjects were psychologists working in government and private healthcare facilities in Saudi Arabia. The World Health Organization Mental Health Atlas 2020 Country Profile for Saudi Arabia reported that the psychologist workforce in the governmental and non-governmental sectors was estimated to be 1113 professionals. A total of 224 psychologists participated in this study. Participants were recruited via convenience sampling, and an online survey was distributed through professional and social networking platforms (e.g., LinkedIn, Twitter, and WhatsApp) between 27 March and 20 April 2023. Eligible participants were psychologists aged 21–60 years who were practicing in Saudi Arabia and were classified and registered with the Saudi Commission for Health Specialties. Undergraduate psychology students, trainees, interns, and individuals residing outside Saudi Arabia at the time of data collection were excluded.

### 2.2. Participant Characteristics

Participants ranged in age from 21 to 60 years. No missing data were identified for the sociodemographic, professional, self-compassion, or psychological flourishing variables included in the analyses; therefore, no imputation or other missing-data procedures were required (Table 1).

### 2.3. Ethical Approval and Informed Consent

The study protocol was approved by the Institutional Review Board (IRB) of Princess Nourah bint Abdulrahman University (approval no. HAP-01-R-059; 21 March 2023). Informed consent was obtained from all the participants before data collection. Participation was voluntary, responses were anonymous, and all procedures were conducted in accordance with the Declaration of Helsinki.

### 2.4. Data Collection

A secure online self-administered questionnaire was designed, and data were collected from 27 March to 20 April 2023. To reach the maximum number of practicing psychologists across Saudi Arabia, the survey link was shared on professional and social networking platforms like LinkedIn, X (formerly Twitter), and WhatsApp. Before accessing the questionnaire, the participants were provided with an electronic information sheet explaining the study objectives, eligibility criteria, voluntary nature of participation, and confidentiality procedures. Informed consent was obtained from all the participants before the questionnaire was initiated. Participants were told that they could withdraw from the study at any time without penalty.

The questionnaire was completed anonymously, and no personally identifiable information was collected. Responses were securely stored and only accessible to the research team. After data collection was completed, the data set was checked for completeness, consistency, and eligibility before statistical analysis.

### 2.5. Measures of Study

#### 2.5.1. Background Information Questionnaire

Participants completed a brief background information form developed for this study, which collected demographic and professional characteristics. The questionnaire included data on age, gender, marital status, professional classification, years of professional experience, and other background characteristics relevant to the study objectives.

#### 2.5.2. Self-Compassion Scale (SCS)

Self-compassion was assessed using the 26-item Self-Compassion Scale (SCS) developed by Neff [19]. The scale comprises six dimensions: self-kindness, common humanity, mindfulness, self-judgment, isolation, and over-identification. Responses were rated on a 5-point Likert scale ranging from 1 (Almost Never) to 5 (Almost Always). For subscale-level correlation and regression analyses, self-judgment, isolation, and over-identification were retained in their original scoring direction, such that higher scores represented greater self-judgment, isolation, and over-identification, respectively. For the total SCS score, negatively worded items were reverse-scored so that higher total scores indicated greater overall self-compassion. Previous research has demonstrated satisfactory psychometric properties of the Arabic version of the SCS, including evidence of reliability and construct validity in Arabic-speaking populations [42].

#### 2.5.3. Flourishing Scale (FS)

Psychological flourishing was assessed using the 8-item Flourishing Scale (FS) developed by Diener et al. [4]. The Arabic version of the Flourishing Scale has demonstrated satisfactory psychometric properties in Saudi samples, including evidence of factorial validity, internal consistency, convergent validity, and discriminant validity [43]. In the present study, the scale was administered in Arabic using a modified 5-point response format ranging from 1 (Strongly Disagree) to 5 (Strongly Agree).

Psychological flourishing was assessed using the 8-item Flourishing Scale (FS) developed by Diener et al. In the present study, the scale was administered in Arabic using a modified 5-point response format ranging from 1 (Strongly Disagree) to 5 (Strongly Agree), yielding a possible total score ranging from 8 to 40. Higher total scores indicate greater psychological flourishing. This response format differs from the original 7-point format of the Flourishing Scale; therefore, psychometric evidence established for the original response format should not be assumed to transfer directly to the modified format. The Flourishing Scale demonstrated good internal consistency in the present sample (Cronbach’s α = 0.90).

In addition, the relatively high mean flourishing score (M = 33.8 out of 40, SD = 3.9), together with the clustering of observed scores toward the upper end of the scale, suggests a possible ceiling effect and restricted outcome variability. This restricted range may have limited the amount of variance available for prediction and, consequently, constrained the attainable R^2^ values across the predictive models. Future studies should consider using measures with greater response-range sensitivity and more heterogeneous samples to better capture variability in psychological flourishing.

#### 2.5.4. Reliability of the Study Measures

These findings indicate adequate internal consistency of the scores obtained from both instruments in the present sample. However, Cronbach’s alpha provides evidence specifically regarding internal consistency and should not be interpreted as evidence of broader psychometric adequacy or measurement validity.

### 2.6. Statistical Analysis

Statistical analyses were conducted using IBM SPSS Statistics version 29.0 (IBM Corp., Armonk, NY, USA) and JASP version 0.19 for descriptive and inferential analyses. The predictive analyses were implemented in Python using scikit-learn version 1.8.0. Descriptive statistics were used to provide an overview of the characteristics of the participants and variables of the study. Internal consistency was determined using Cronbach’s alpha. To explore the relationships between the dimensions of self-compassion and psychological flourishing, Pearson’s correlation analysis was conducted. Multiple linear regression was performed to examine the independent associations between the self-compassion dimensions and psychological flourishing.

Regression diagnostics were examined prior to interpreting the multiple linear regression model. Linearity, residual distribution, homoscedasticity, multicollinearity, and influential observations were assessed. Variance inflation factors ranged from 1.60 to 3.15, indicating no problematic multicollinearity, and the maximum Cook’s distance was 0.075, indicating no highly influential observations. Because diagnostic testing indicated some departure from residual normality (Shapiro–Wilk *p* = 0.014) and evidence of heteroscedasticity (Breusch–Pagan *p* = 0.028), HC3 heteroscedasticity-consistent robust standard errors were used for statistical inference. To complement the traditional statistical analyses, a reproducible supervised machine-learning procedure was implemented to predict psychological flourishing. The predictor set comprised the six self-compassion dimensions together with gender, age group, marital status, professional classification, and years of professional experience. The dataset was randomly partitioned into a training set (80%; n = 179) and an independent held-out test set (20%; n = 45), using a fixed random seed of 42. Categorical predictors were dummy encoded, and continuous predictors were standardized where required by the algorithm. Hyperparameter selection was conducted exclusively within the training set using five-fold cross-validation. For Random Forest, the hyperparameter search space included estimators = {100, 200, 500}, max_features = {sqrt, log2, 1.0} [33], and min_samples_leaf = {1, 2, 4}. For SVR, the search space included C = {1, 10, 100}, gamma = {0.001, 0.01, 0.1}, and epsilon = {0.10, 0.25, 0.50}. Hyperparameter configurations were compared using the root mean square error (RMSE) averaged across the five validation folds, with lower RMSE indicating better cross-validated predictive performance. All preprocessing steps were incorporated within the cross-validation workflow to prevent information leakage. For SVR, standardization parameters were estimated separately from the training portion of each cross-validation fold and subsequently applied to the corresponding validation portion; the held-out test set remained completely excluded from preprocessing, model fitting, and hyperparameter selection.

Random Forest Regression and Support Vector Regression (SVR) with a radial basis function (RBF) kernel were evaluated. For Random Forest, the tuning procedure selected 1000 trees, the square root of the available features at each split, and a minimum leaf size of 2. For SVR, the selected hyperparameters were C = 100, gamma = 0.001, and epsilon = 0.50. The held-out test set was not used during model training or hyperparameter selection. Final predictive performance was evaluated exclusively on the held-out test set using the coefficient of determination (R^2^), root mean square error (RMSE), and mean absolute error (MAE). The same training–test partition and predictor set were also used for linear regression to permit a like-for-like comparison across models.

## 3. Results

### 3.1. What Are the Associations Between Overall Self-Compassion, Its Six Dimensions, and Psychological Flourishing Among Psychologists in Saudi Arabia?

The Pearson correlation analysis showed a significant positive correlation between total self-compassion and psychological flourishing among psychologists (r = 0.627, *p* < 0.001; Table 2). For the positive dimensions of self-compassion, the strongest positive correlation was found for self-kindness (r = 0.557, *p* < 0.001), followed by mindfulness (r = 0.410, *p* < 0.001) and common humanity (r = 0.302, *p* < 0.001). In contrast, non-compassionate aspects were significantly and negatively related to psychological flourishing, including self-judgment (r = −0.524, *p* < 0.001), over-identification (r = −0.504, *p* < 0.001), and isolation (r = −0.465, *p* < 0.001). In summary, these findings indicate that psychologists with higher levels of self-compassion are likely to experience greater psychological flourishing.

### 3.2. Which Dimensions of Self-Compassion Are Independently Associated with Psychological Flourishing Among Psychologists When Examined Using Traditional Multiple Regression Analysis?

Multiple linear regression analysis was conducted to examine the independent associations between the six self-compassion dimensions and psychological flourishing. The overall model was statistically significant, F(6, 217) = 24.61, *p* < 0.001, explaining 40.5% of the variance in psychological flourishing (R^2^ = 0.405, adjusted R^2^ = 0.388). Self-kindness showed a significant positive independent association with psychological flourishing (β = 0.292, *p* = 0.001), whereas over-identification showed a significant negative independent association (β = −0.206, *p* = 0.009). Common humanity, mindfulness, self-judgment, and isolation were not independently associated with psychological flourishing after adjustment for the remaining dimensions.

Although variance inflation factors did not indicate problematic multicollinearity, the relatively strong correlations among some self-compassion dimensions indicate substantial shared variance; therefore, the unique regression coefficients for individual dimensions should be interpreted cautiously. As shown in Table 3, the overall model was statistically significant, F(6, 217) = 24.61, *p* < 0.001, explaining 40.5% of the variance in psychological flourishing (R^2^ = 0.405, adjusted R^2^ = 0.388).

### 3.3. What Is the Predictive Performance of Machine-Learning Models in Predicting Psychological Flourishing Among Psychologists Using Self-Compassion Dimensions and Demographic/Professional Characteristics?

**Figure 1:**
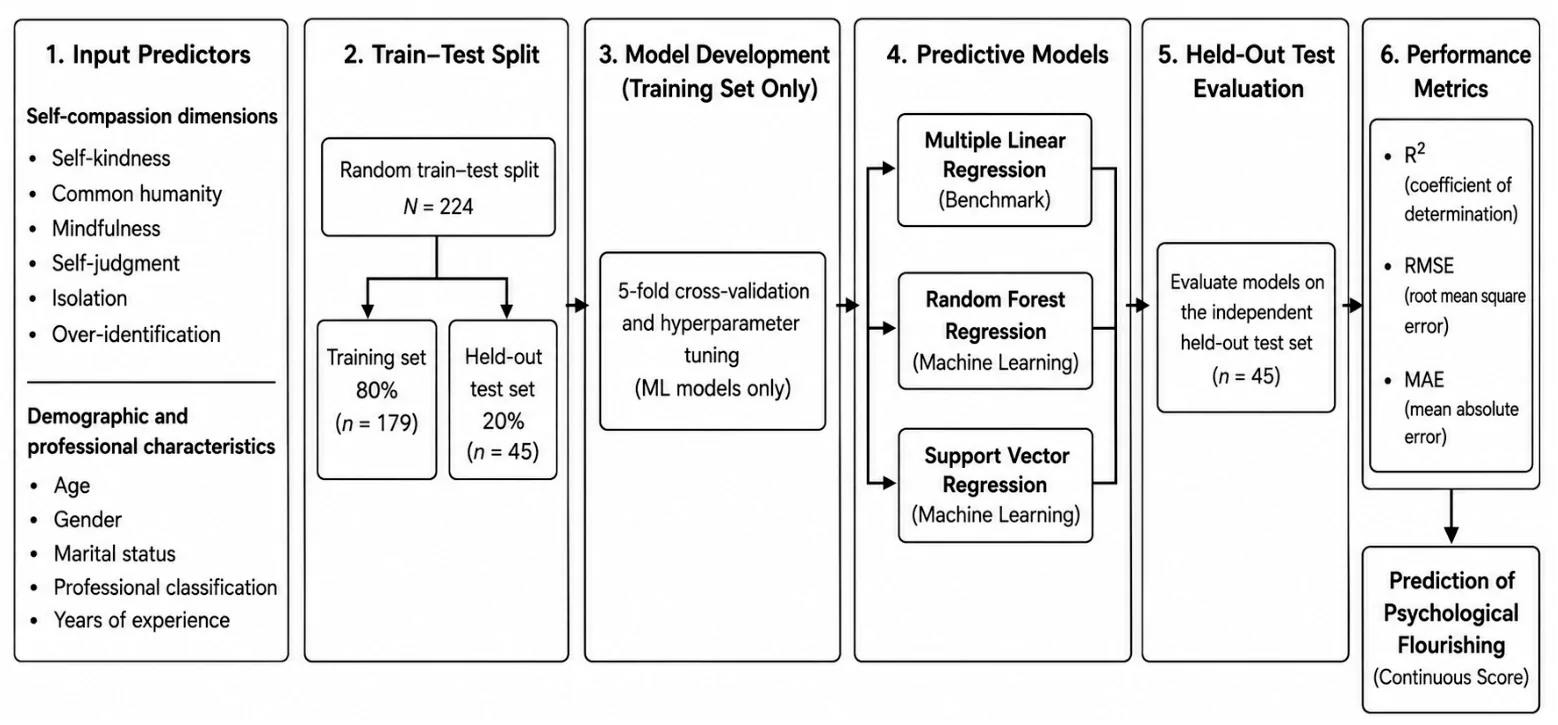
Predictive modeling workflow for psychological flourishing among psychologists. The dataset (N = 224) was randomly partitioned into a training set (80%; n = 179) and an independent held-out test set (20%; n = 45). Model development and hyperparameter tuning for the machine-learning models were conducted exclusively within the training set using five-fold cross-validation. Multiple Linear Regression was included as the linear benchmark, alongside Random Forest Regression and Support Vector Regression. All three models were evaluated on the same independent held-out test set using the coefficient of determination (R^2^), root mean square error (RMSE), and mean absolute error (MAE) to predict psychological flourishing as a continuous outcome.

The third research question examined the predictive performance of two supervised machine-learning algorithms, Random Forest Regression and Support Vector Regression (SVR), using the six self-compassion dimensions together with demographic and professional characteristics. Both models were evaluated on the same independent held-out test set (n = 45) following model training and hyperparameter selection using the training data (Figure 1). Random Forest Regression yielded a test-set R^2^ of 0.282, with an RMSE of 3.562 and an MAE of 2.771. SVR yielded a test-set R^2^ of 0.272, with an RMSE of 3.587 and an MAE of 2.768. Thus, the two machine-learning models demonstrated broadly comparable and modest out-of-sample predictive performance, with Random Forest showing a slightly higher R^2^ and lower RMSE, whereas SVR showed a marginally lower MAE. The comparative held-out test-set performance of the machine-learning models and the linear regression benchmark is presented in Table 4.

### 3.4. How Does the Predictive Performance of Machine Learning Models Compare with That of Traditional Multiple Linear Regression in Predicting Psychological Flourishing Among Psychologists?

**Figure 2:**
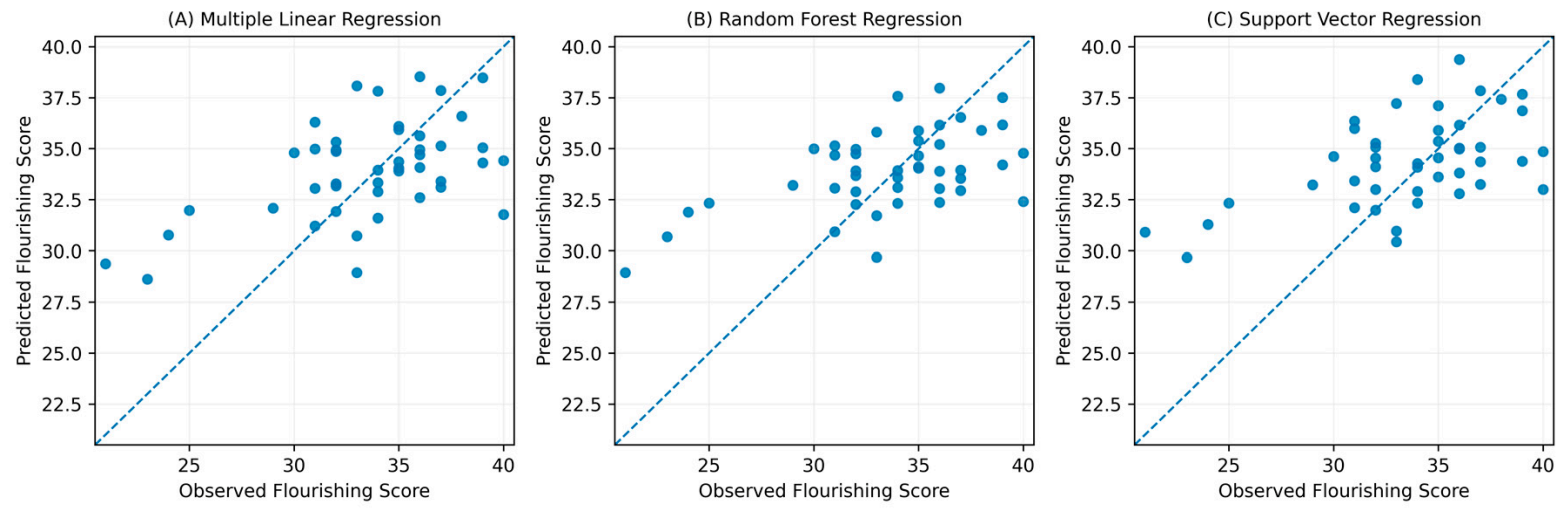
Observed versus predicted psychological flourishing scores on the independent held-out test set for (**A**) Multiple Linear Regression, (**B**) Random Forest Regression, and (**C**) Support Vector Regression. The dashed diagonal line represents perfect agreement between observed and predicted scores. As shown in Figure 3, the residual plots illustrate the distribution of prediction errors across the three models on the held-out test set.

For the fourth research question, the predictive performance of multiple linear regression, Random Forest Regression, and Support Vector Regression was compared using an identical predictor set and the same held-out test partition. For this predictive comparison, all three models included the six self-compassion dimensions together with gender, age group, marital status, professional classification, and years of professional experience. On the independent held-out test set (n = 45), multiple linear regression yielded an R^2^ of 0.273, an RMSE of 3.583, and an MAE of 2.849. Random Forest Regression yielded an R^2^ of 0.282, an RMSE of 3.562, and an MAE of 2.771, whereas SVR yielded an R^2^ of 0.272, an RMSE of 3.587, and an MAE of 2.768. Overall, predictive performance was highly similar across the three approaches. Random Forest achieved the highest test-set R^2^ and the lowest RMSE, while SVR produced the lowest MAE by a small margin. These findings provide no evidence of a substantial predictive advantage of the machine-learning models over the linear benchmark in the present sample. As shown in Figure 2, the observed versus predicted psychological flourishing scores on the independent held-out test set illustrate the predictive performance of the three models.

**Figure 3:**
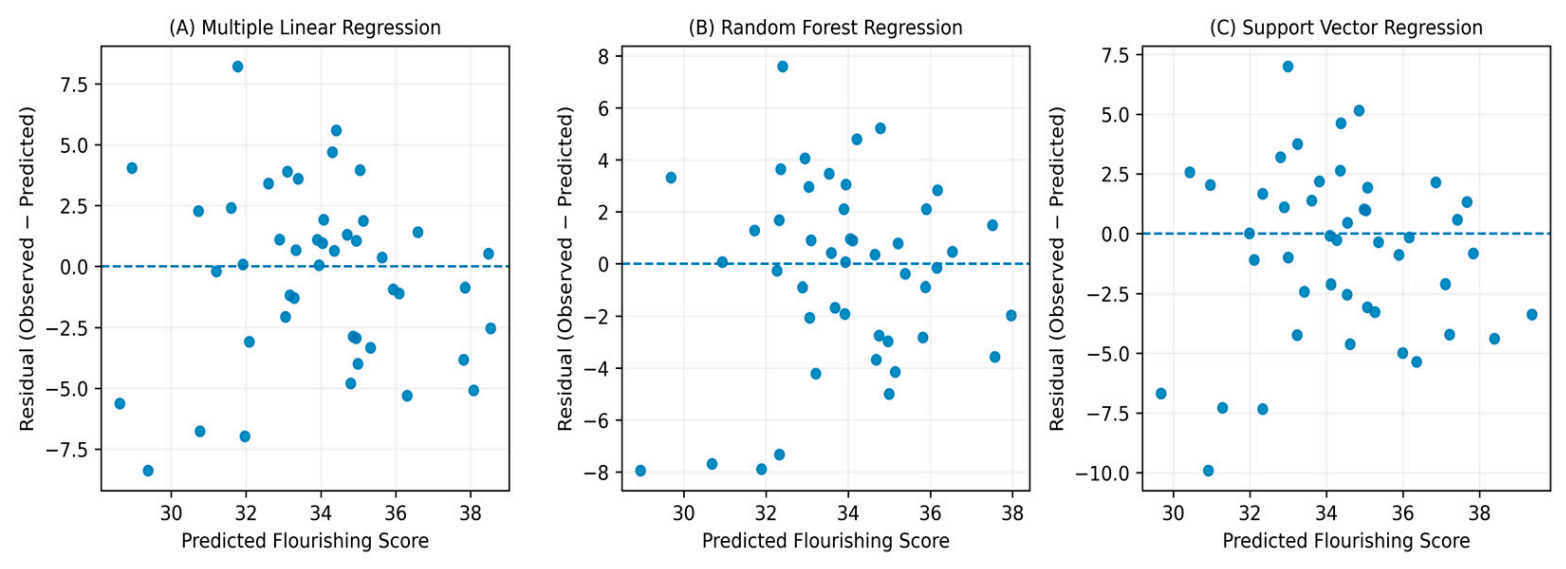
Residual plots for held-out test-set predictions from (**A**) Multiple Linear Regression, (**B**) Random Forest Regression, and (**C**) Support Vector Regression. Residuals were calculated as observed minus predicted psychological flourishing scores, with the dashed horizontal reference line at zero indicating perfect prediction.

## 4. Discussion

This study examined the relationship between self-compassion and psychological flourishing among psychologists in Saudi Arabia using traditional statistical and supervised machine learning approaches. The findings are discussed in relation to four research questions addressing the association between self-compassion and flourishing, the unique contribution of self-compassion dimensions, the predictive performance of machine learning models, and their comparison with traditional multiple linear regression.

### 4.1. Relationship Between Self-Compassion and Psychological Flourishing

The present study found a significant positive association between total self-compassion and psychological flourishing among psychologists in Saudi Arabia (r = 0.627, *p* < 0.001). Thus, psychologists who reported higher levels of self-compassion also reported higher levels of flourishing. The size of the association suggests that self-compassion is an important psychological resource and not a trivial correlate of well-being.

Recent evidence similarly supports a positive association between self-compassion and psychological flourishing. For example, Liu et al. [26], in a study of 842 university students, reported a significant positive association between self-compassion and psychological flourishing and further identified hope and emotion regulation as important mechanisms involved in this relationship. These findings are broadly consistent with the positive association observed in the present study, although differences in population and study design should be considered.

The highest positive correlation was between self-kindness and flourishing (r = 0.557, *p* < 0.001), followed by mindfulness and common humanity, as revealed by dimensional analysis. This pattern is theoretically consistent with the notion that compassionate self-responding facilitates adaptive emotion regulation, reduces harsh self-judgment, and enables individuals to maintain a balanced perspective during stressful experiences. Recent systematic evidence also shows that self-compassion is linked to improvements in mental health through decreases in negative repetitive thinking and increases in adaptive coping and emotion regulation [25]. Several interventions to promote compassion in health professionals have shown positive effects on self-compassion and occupational well-being, illustrating the practical relevance of these psychological processes for demanding helping professions. Conversely, self-judgment, over-identification, and isolation were negatively associated with psychological flourishing. The findings indicate that psychologists who respond to professional or personal problems with high levels of self-criticism, emotional overinvolvement, or perceived isolation may have less than optimal levels of psychological functioning. This pattern is in line with contemporary conceptualizations of self-compassion as a process that involves both compassionate and uncompassionate responses, which are different but related, with differential implications for mental health. Recently, self-compassion training has been suggested as a potential intervention for enhancing well-being and reducing psychological distress [25,27]. However, the magnitude of these effects can be context- and sample-dependent. These findings are particularly important for psychologists who, through their work, are repeatedly exposed over time to emotionally taxing interpersonal situations.

Thus, the strong association observed in the present study suggests that self-compassion may represent a relevant psychological resource for professional well-being among psychologists. However, given the cross-sectional design, the directionality and causality of this association cannot be established.

### 4.2. Independent Associations of Self-Compassion Dimensions with Psychological Flourishing

This study demonstrated that the six dimensions of self-compassion were not equally associated with psychological flourishing when examined simultaneously. Self-kindness showed a significant positive independent association with psychological flourishing, whereas over-identification showed a significant negative independent association. In contrast, common humanity, mindfulness, self-judgment, and isolation were not independently associated with psychological flourishing after accounting for shared variance among the self-compassion dimensions. These findings suggest that some dimensions of self-compassion are more strongly associated with psychological flourishing than others. This is consistent with previous research on the benefits of self-kindness and the notion that empathy, warmth, and acceptance of self in times of stress may relate to adaptive emotion regulation, resilience, and psychological well-being.

Higher self-kindness may help psychologists cope more adaptively with professional demands by reducing harsh self-evaluation and supporting emotional regulation and positive psychological functioning.

Conversely, the negative independent association of over-identification is consistent with evidence linking excessive engagement with negative thoughts and emotions to poorer psychological well-being [44]. Interestingly, the other dimensions were significantly correlated with bivariate psychological flourishing but did not show significant independent associations in the multivariable regression model. This finding is not unexpected, as the six dimensions represent conceptually related aspects of the broader self-compassion construct and therefore share substantial variance. When examined simultaneously within a multivariable regression model, only the dimensions explaining the unique variance remained statistically significant. Accordingly, future intervention studies could examine whether strategies designed to enhance self-kindness and reduce over-identification contribute to improvements in psychological flourishing among psychologists. Nevertheless, these findings should be interpreted cautiously because the cross-sectional design does not permit conclusions regarding causal relationships.

### 4.3. Machine Learning Prediction of Psychological Flourishing

In addition to the traditional statistical analyses, two supervised machine-learning algorithms, Random Forest Regression and Support Vector Regression (SVR), were used to predict psychological flourishing from the six self-compassion dimensions together with demographic and professional characteristics. When evaluated on the independent held-out test set, both machine-learning models showed modest out-of-sample predictive performance. Random Forest Regression yielded an R^2^ of 0.282, an RMSE of 3.562, and an MAE of 2.771, whereas SVR yielded an R^2^ of 0.272, an RMSE of 3.587, and an MAE of 2.768. The small differences across performance metrics indicate that neither machine-learning algorithm consistently outperformed the other. Specifically, the linear regression benchmark yielded R^2^ = 0.273, RMSE = 3.583, and MAE = 2.849 on the same held-out test set. This finding suggests that, within the present sample and predictor space, the added algorithmic complexity of Random Forest and SVR did not produce a substantial improvement in out-of-sample prediction. Model performance may depend on characteristics such as sample size, predictor structure, and the underlying complexity of predictor–outcome relationships [38,39]. Accordingly, the present findings support a cautious interpretation of the machine-learning results rather than evidence of clear predictive superiority over conventional linear modeling.

The comparable performance of the linear and machine-learning models should also be considered in relation to sample size and validation scope. Recent methodological evidence indicates that predictive performance can become less stable in relatively small samples and that increasing model complexity does not necessarily improve generalization under such conditions. Moreover, performance established through internal validation or a held-out subset from the same dataset should not be equated with external generalizability, which requires evaluation in independent populations or settings. These considerations reinforce the need to interpret the present machine-learning findings cautiously and to examine their reproducibility in larger, independent samples [28,30].

Given the modest out-of-sample performance observed in the present study, the machine-learning findings should be interpreted primarily as preliminary evidence of feasibility rather than as evidence of clinical predictive utility. The models illustrate the potential for integrating self-compassion, demographic, and professional characteristics within predictive frameworks; however, their current performance does not support individual-level screening or clinical decision-making. Machine-learning approaches should therefore be viewed as complementary analytical tools rather than replacements for professional psychological assessment and clinical judgment. Future studies should use larger and more diverse samples, repeated or nested validation procedures, and external validation in independent samples to determine whether predictive performance and generalizability can be improved.

The present findings are also consistent with the broader methodological literature emphasizing rigorous validation when machine-learning models are applied to health-related data. Recent studies have demonstrated the value of comparing multiple algorithms under consistent validation procedures and have highlighted the importance of preprocessing, cross-validation, and independent or subject-disjoint evaluation for estimating model generalizability. Such considerations are particularly important when predictors are derived from self-reported psychological measures, for which response errors may affect model stability. Accordingly, the relatively similar out-of-sample performance of linear regression, Random Forest, and SVR in the present study should not be interpreted as evidence of clinical utility or superiority of a particular algorithm, but rather as preliminary evidence that nonlinear models did not provide a substantial predictive advantage within the present sample and predictor set [38,41,45].

### 4.4. Comparison Between Traditional Regression and Machine Learning Models

The comparison between traditional multiple linear regression and the two machine-learning models was conducted using the same predictor set and the same independent held-out test partition, thereby allowing a direct comparison of out-of-sample predictive performance. Performance was highly similar across the three approaches. Multiple linear regression yielded an R^2^ of 0.273, an RMSE of 3.583, and an MAE of 2.849. Random Forest Regression yielded an R^2^ of 0.282, an RMSE of 3.562, and an MAE of 2.771, whereas Support Vector Regression yielded an R^2^ of 0.272, an RMSE of 3.587, and an MAE of 2.768. Random Forest therefore achieved the highest test-set R^2^ and the lowest RMSE, while SVR achieved the lowest MAE by a small margin. However, the differences among the three models were small and do not indicate a meaningful predictive advantage of either machine-learning approach over the linear benchmark.

These findings are informative because they distinguish the inferential role of the full-sample multiple regression analysis from the predictive comparison conducted on held-out data. The former was used to examine the independent statistical contributions of the six self-compassion dimensions, whereas the latter evaluated out-of-sample prediction using an identical set of self-compassion, demographic, and professional predictors across all three models. The comparable test-set performance suggests that the relationships captured by the available predictors may not require substantially greater algorithmic complexity in the present sample. Accordingly, Random Forest and SVR should be viewed as complementary predictive approaches rather than as demonstrably superior alternatives to linear regression. Further research using larger independent samples and external validation is needed to determine whether machine-learning models provide incremental predictive value beyond conventional regression approaches [24,39,40].

## 5. Limitations and Strengths

The strengths of the current study are as follows: First, it builds on prior literature by integrating classic multiple linear regression and supervised machine learning approaches in the study of psychological flourishing in psychologists, thus combining approaches of explanatory and predictive analytics. Second, the study employed widely used psychometric measures and demonstrated adequate internal consistency for both measures in the present sample. Third, the study focuses on practicing psychologists, an occupational group that has been relatively ignored despite their critical role in promoting mental health. The final comparison of traditional statistical approaches and models based on machine learning offers a more holistic view of the factors associated with psychological flourishing and the potential value of predictive analytics in positive psychology research.

Nevertheless, several limitations of this study should be acknowledged. First, the cross-sectional design of the study did not allow causal inference of the associations observed. Second, self-report measures might have introduced the possibility of common method bias and social desirability bias. Third, the Flourishing Scale was administered using a modified 5-point response format rather than its original 7-point format. Although the scale demonstrated good internal consistency in the present sample, this modification may affect comparability with studies using the original response format and should be considered when interpreting the findings. In addition, the relatively high mean flourishing score (M = 33.79 out of 40, SD = 3.89), together with the clustering of scores toward the upper end of the scale, suggests a possible ceiling effect and restricted outcome variability. This restricted range may have limited the variance available for prediction and consequently constrained the attainable R^2^ values across the predictive models. Future studies should consider measures with greater response-range sensitivity and more heterogeneous samples to capture a broader range of psychological flourishing. Finally, although the machine learning models demonstrated modest out-of-sample predictive performance, their accuracy and generalizability should be interpreted cautiously given the relatively small sample size and the absence of external validation using an independent dataset.

In addition, the unequal representation of some demographic and professional subgroups, including the predominance of women and the small number of participants in certain professional categories, may have limited the models’ ability to learn stable subgroup-specific patterns. This sample composition should therefore be considered when interpreting the predictive estimates and their generalizability to the broader population of psychologists. Moreover, despite the use of a held-out test set and cross-validation within the training data, the possibility of model overfitting cannot be entirely excluded, and the reported predictive estimates remain dependent on the internal validation strategy used in this study.

## 6. Practical Implications

The present findings may have implications for psychological practice, professional training, and mental health policy. Self-kindness and over-identification emerged as dimensions independently associated with psychological flourishing, suggesting that these components may warrant consideration in the development and evaluation of future self-compassion-based interventions. Accordingly, future intervention studies could examine whether incorporating self-compassion strategies into graduate education, continuing professional development activities, and workplace well-being programs may contribute to psychological flourishing, occupational stress management, resilience, and professional functioning among psychologists. Moreover, the modest out-of-sample predictive performance observed in the present study suggests that machine-learning approaches may serve as exploratory complements to traditional statistical analyses rather than as established tools for individual-level prediction. Given the absence of a substantial predictive advantage over linear regression and the lack of external validation, the present models should not be interpreted as supporting clinical screening or decision-making. Further validation in larger and independent samples is required before any practical predictive application can be considered.

## 7. Conclusions

This study examined the association between self-compassion and psychological flourishing among psychologists in Saudi Arabia using traditional statistical and supervised machine-learning approaches. Self-compassion was positively associated with psychological flourishing. In the traditional multiple regression analysis, self-kindness showed a significant positive independent association with psychological flourishing, whereas over-identification showed a significant negative independent association. In the predictive comparison, multiple linear regression, Random Forest Regression, and Support Vector Regression were evaluated using the same predictor set and the same independent held-out test partition. Their out-of-sample predictive performance was highly similar, with Random Forest achieving the highest test-set R^2^ and lowest RMSE, while SVR achieved the lowest MAE by a small margin. Importantly, neither machine-learning models demonstrated a substantial predictive advantage over the linear regression benchmark. These findings suggest that, within the present sample and predictor set, greater algorithmic complexity did not translate into meaningfully improved prediction. Machine-learning methods may nevertheless provide complementary analytical perspectives, but their predictive utility requires further evaluation using larger independent samples and external validation. Collectively, the findings highlight self-compassion, particularly self-kindness and lower over-identification, as a psychological characteristic associated with flourishing among psychologists and provide a basis for future longitudinal and intervention research examining strategies to support psychological flourishing and professional well-being.

## Figures and Tables

**Table 1:** Demographic and professional characteristics of the study participants (N = 224).

Characteristic	Total (N = 224)
Age (years)	
21–30	112 (50.0%)
31–40	73 (32.6%)
41–50	31 (13.8%)
51–60	8 (3.6%)
Gender	
Male	65 (29.0%)
Female	159 (71.0%)
Marital status	
Single	116 (51.8%)
Married	93 (41.5%)
Divorced	15 (6.7%)
Professional classification	
Assistant psychologist	68 (30.4%)
Specialist psychologist	110 (49.1%)
Senior psychologist	41 (18.3%)
Consultant psychologist	5 (2.2%)
Professional experience (years)	
0–3	79 (35.3%)
3–7	74 (33.0%)
≥8	71 (31.7%)

**Table 2:** Descriptive statistics and Pearson correlation matrix of psychological flourishing, self-compassion, and its dimensions.

Variable	Mean ± SD	1	2	3	4	5	6	7	8
1. Psychological Flourishing	33.79 ± 3.89	—							
2. Total Self-Compassion	94.71 ± 14.34	0.627 ***	—						
3. Self-Kindness	19.25 ± 3.74	0.557 ***	0.839 ***	—					
4. Common Humanity	14.65 ± 3.12	0.302 ***	0.521 ***	0.505 ***	—				
5. Mindfulness	14.91 ± 2.53	0.410 ***	0.724 ***	0.655 ***	0.479 ***	—			
6. Self-Judgment	12.26 ± 3.69	−0.524 ***	−0.828 ***	−0.568 ***	−0.154 *	−0.406 ***	—		
7. Isolation	9.96 ± 3.14	−0.465 ***	−0.752 ***	−0.487 ***	−0.098	−0.342 ***	0.705 ***	—	
8. Over-identification	9.87 ± 3.05	−0.504 ***	−0.762 ***	−0.470 ***	−0.076	−0.381 ***	0.764 ***	0.670 ***	—

Note. Values are presented as mean ± SD. Pearson correlation coefficients are reported. * *p* < 0.05, *** *p* < 0.001.

**Table 3:** Multiple linear regression examining the independent associations between self-compassion dimensions and psychological flourishing (N = 224).

Predictor	B	SE (HC3)	β	t	*p*	95% CI for B	Tolerance	VIF
Self-kindness	0.303	0.093	0.292	3.248	0.001	[0.119, 0.487]	0.407	2.46
Common humanity	0.137	0.084	0.110	1.620	0.107	[−0.030, 0.303]	0.625	1.60
Mindfulness	0.005	0.122	0.003	0.044	0.965	[−0.235, 0.246]	0.499	2.01
Self-judgment	−0.126	0.095	−0.120	−1.326	0.186	[−0.314, 0.061]	0.317	3.15
Isolation	−0.110	0.101	−0.089	−1.095	0.275	[−0.309, 0.088]	0.451	2.22
Over-identification	−0.263	0.099	−0.206	−2.654	0.009	[−0.458, −0.068]	0.371	2.70

Note. B = unstandardized regression coefficient; SE = HC3 heteroscedasticity-consistent robust standard error; CI = confidence interval; VIF = variance inflation factor. Model statistics: R^2^= 0.405, adjusted R^2^ = 0.388, F(6, 217) = 24.61, *p* < 0.001, Durbin–Watson = 1.99.

**Table 4:** Comparative Held-Out Test-Set Performance of Linear Regression and Machine-Learning Models for Predicting Psychological Flourishing.

Model	Test-Set R^2^	RMSE	MAE
Multiple Linear Regression	0.273	3.583	2.849
Random Forest Regression	0.282	3.562	2.771
Support Vector Regression	0.272	3.587	2.768

Note. R^2^ = coefficient of determination; RMSE = root mean square error; MAE = mean absolute error. All three models were fitted using the same predictor set and evaluated on the same independent held-out test set (n = 45). Higher R^2^ and lower RMSE and MAE indicate better predictive performance.

## Data Availability

The data are not publicly available due to ethical, confidentiality, and institutional data-governance requirements. Access to the data is subject to prior authorization from the relevant institutional authority and compliance with the conditions of the ethical approval.
